# Transcutaneous auricular vagus nerve stimulation improves gait and cortical activity in Parkinson's disease: A pilot randomized study

**DOI:** 10.1111/cns.14309

**Published:** 2023-06-13

**Authors:** Heng Zhang, Xing‐yue Cao, Li‐na Wang, Qing Tong, Hui‐min Sun, Cai‐ting Gan, Ai‐di Shan, Yong‐sheng Yuan, Ke‐zhong Zhang

**Affiliations:** ^1^ Department of Neurology The First Affiliated Hospital of Nanjing Medical University Nanjing China

**Keywords:** functional near‐infrared spectroscopy, gait impairments, Parkinson's disease, primary somatosensory cortex, transcutaneous auricular vagus nerve stimulation

## Abstract

**Objective:**

In this randomized, double‐blind, sham‐controlled trial, we explored the effect of 20 Hz transcutaneous auricular vagus nerve stimulation (taVNS) on gait impairments in Parkinson's disease (PD) patients and investigated the underlying neural mechanism.

**Methods:**

In total, 22 PD patients and 14 healthy controls were enrolled. PD patients were randomized (1:1) to receive active or sham taVNS (same position as active taVNS group but without releasing current) twice a day for 1 week. Meanwhile, all subjects were measured activation in the bilateral frontal and sensorimotor cortex during usual walking by functional near‐infrared spectroscopy.

**Results:**

PD patients showed instable gait with insufficient range of motion during usual walking. Active taVNS improved gait characteristics including step length, stride velocity, stride length, and step length variability compared with sham taVNS after completion of the 7‐day therapy. No difference was found in the Unified Parkinson's Disease Rating Scale III, Timed Up and Go, Tinetti Balance, and Gait scores. Moreover, PD patients had higher relative change of oxyhemoglobin in the left dorsolateral prefrontal cortex, pre‐motor area, supplementary motor area, primary motor cortex, and primary somatosensory cortex than HCs group during usual walking. Hemodynamic responses in the left primary somatosensory cortex were significantly decreased after taVNS therapy.

**Conclusion:**

taVNS can relieve gait impairments and remodel sensorimotor integration in PD patients.

## Graphical abstract

The 20 Hz transcutaneous auricular vagus nerve stimulation (taVNS) could relieve gait impairments and remodel sensorimotor integration in PD patients. The results provided insights into the neural mechanism of taVNS and a new neuromodulation method for treating gait impairments in PD patients.

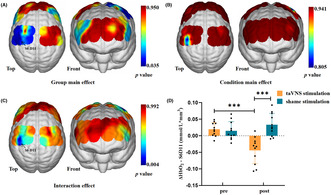

## 1 INTRODUCTION

Parkinson's disease (PD), one of the most common neurodegenerative diseases, conveys a mounting socioeconomic burden.^1^ Gait impairments arising from the typical pathophysiological manifestations of PD are among its most recurring and disabling symptoms.^2^ Early intervention for PD patients can increase their benefits as gait impairments progress with the process of the disease.^2^ However, the primary treatment of gait disorders in PD is still dopaminergic drug therapy to date. Dopaminergic medications that improve the speed and amplitude of gait might also pose various challenges that can further damage gait.^3^ Thus, it is imperative to find practical, non‐pharmacological interventions that could ameliorate gait impairments in PD patients.

Vagus nerve stimulation (VNS), performed by a surgically implantable device, has been approved by the Food and Drug Administration as an adjunct neuromodulation therapy for drug‐resistant epilepsy^4^ and depression^5^ for decades. Intriguing piece of evidence from animal models of PD revealed that VNS was beneficial to improving locomotion.^6^, ^7^, ^8^, ^9^, ^10^ In addition, two independent preliminary studies showed that a single application of cervical non‐invasive VNS (nVNS) could improve the gait of PD patients.^11^, ^12^ A randomized sham‐controlled crossover study revealed that 1 month of treatment with cervical nVNS could significantly ameliorate the motor function and gait of PD patients with freezing of gait (FOG).^13^ Altogether, these studies above provided evidence for the efficacy of nVNS on gait impairment in PD. Recently, a pilot‐controlled study found that transcutaneous auricular VNS (taVNS) could be a valuable tool for neuromodulation in PD.^14^ Meanwhile, intrinsic auricular muscle zones (IAMZs) electrical stimulation which allows the potential to provide muscle feedback, modulate motor driver cortical areas, and stimulate multiple nerves including vagus nerve synchronously could improve the clinical motor symptoms^15^ and gait parameters^16^ of PD patients in the short term. Consequently, we used taVNS, a safer and better‐tolerated non‐invasive method of VNS through stimulating the auricular branch of the vagus nerve,^17^ to verify its efficacy and safety of gait impairment in PD patients.

The clinical efficacy of nVNS wins growing recognition in neurological disorders, while its underlying mechanism remains elusive. Given that taVNS could induce widespread, diffuse cortical effects through nucleus tractus solitarius (NTS) and locus coeruleus (LC),^18^, ^19^ it is beneficial for us to further explore the functional alteration of the cerebral cortex to identify the action of nVNS. Mobile functional near‐infrared spectroscopy (fNIRS) is a non‐invasive imaging device that measures hemodynamic alterations of the cerebral cortex induced by local neural activity (according to neurovascular coupling), Which is analogous to functional magnetic resonance imaging but more portable and flexible.^20^ The effectiveness of fNIRS in detecting hemodynamic alterations during active walking has been highlighted in recent documents.^21^, ^22^ Furthermore, accumulating fNIRS studies have reported that the prefrontal and sensorimotor cortex activity is higher during steady‐state walking in PD patients compared with healthy controls, which might compensate for the reduced gait automaticity.^23^, ^24^, ^25^ Hence, our study examined prefrontal and sensorimotor cortex activity during usual walking in PD patients before and after taVNS treatment to further understand the mechanism of taVNS therapies.

## 2 METHODS

### 2.1 Study design and participants

This was a pilot, randomized, double‐blind, and sham‐controlled study (registration no. NCT05561348). Eligible PD patients were recruited and then randomly (1:1) assigned to the taVNS stimulation group or sham stimulation group. A researcher who did not participate in the evaluations and statistical analysis used SPSS v25.0 software (IBM) to get the random number and was responsible for the interventions for two groups of patients. Our study was approved by the ethics committee of the First Affiliated Hospital of Nanjing Medical University (2022‐SR‐535). Written informed consent was signed by all participants prior to the study. Investigators and all PD patients were blinded to the interventions during the study.

Right‐handed patients with idiopathic PD, visiting the Neurology Department of the First Affiliated Hospital of Nanjing Medical University were enrolled. The following inclusion criteria should be satisfied: (1) meet the diagnostic criteria of idiopathic PD according to the Movement Disorder Society (MDS) Clinical Diagnostic Criteria for PD^26^; (2) with the ability to walk for at least 1 min and turn 180° unassisted; (3) Hoehn and Yahr (H&Y)^27^ stage ≤2 in ON medication state; (4) stable dopaminergic therapy at least 4 weeks prior to the study; and (5) 40–80 years old. Simultaneously, participants who met any of the following exclusion criteria were eliminated: (1) with cognitive impairment, according to Mini‐Mental State Examination (MMSE) <24; (2) with current intake of anticholinergics or any drugs that could induce cerebral functional change; (3) with taVNS contraindications, such as implanted cardiac pacemaker or treatment with deep brain stimulation; (4) with known or suspected cardiovascular disease, uncontrolled hypertension or recent myocardial infarction; and (5) with unavoidable factors affecting gait, like osteoarthritis, musculoskeletal disorder, severe visual impairment. Meanwhile, 14 age‐, sex‐, and education‐matched right‐handed healthy controls (HCs) were recruited to provide the baseline assessment data to evaluate the severity of motor/non‐motor symptoms and alterations in the cerebral cortex activity in the PD patient. Participants who had cognitive impairment or any disease affecting gait were excluded.

### 2.2 Study procedure and intervention

**FIGURE 1:**
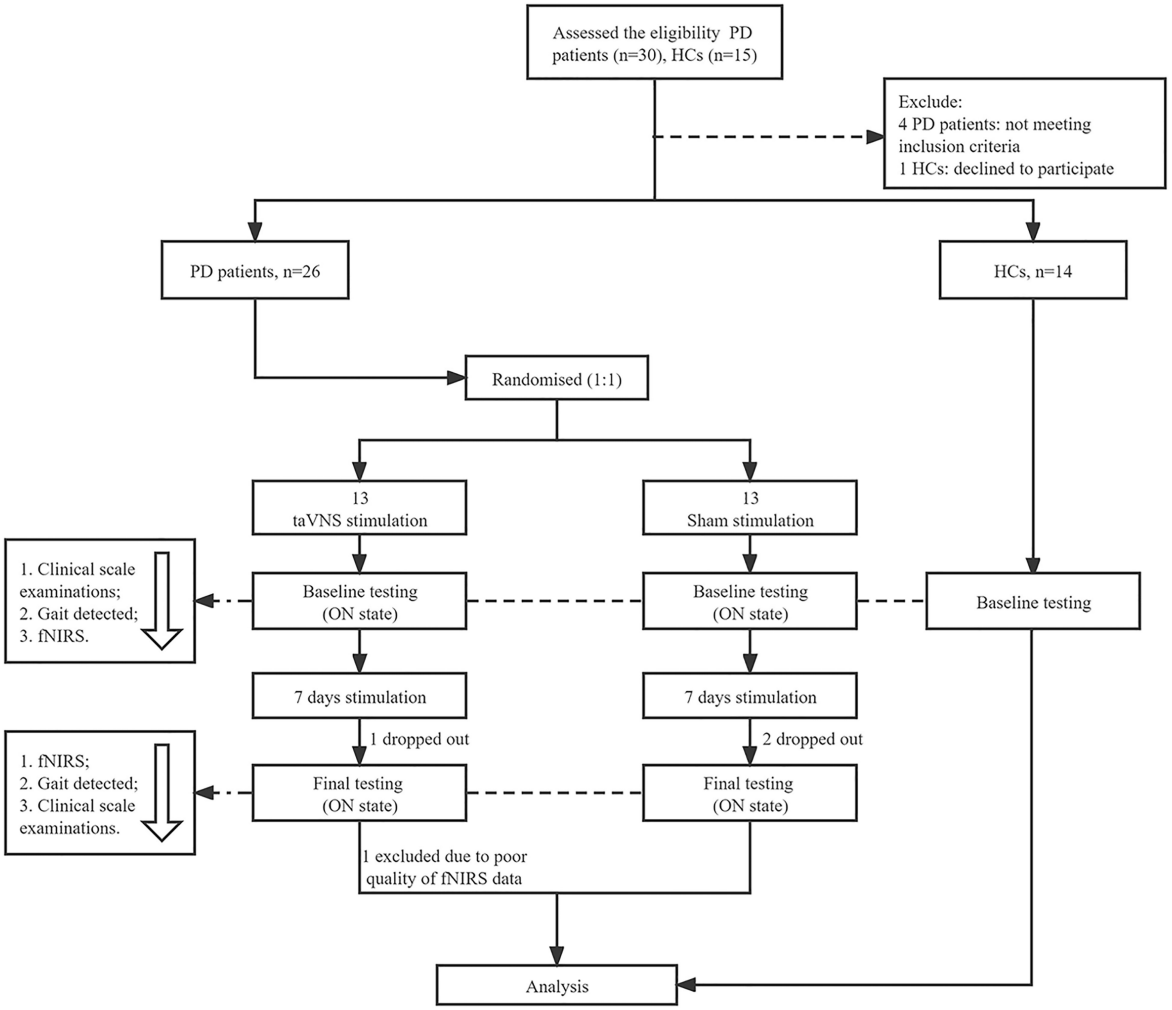
Flow diagram. fNIRS, functional near‐infrared spectroscopy; HCs, healthy control; PD, Parkinson's disease; taVNS, transcutaneous auricular vagus nerve stimulation.

The study flow is shown in Figure 1. At the screening visit, all subjects were reviewed about their age, education, disease duration history, initial side of onset of motor symptoms, and medications, and the eligibility was accessed according to inclusion and exclusion criteria, prior to randomization. Meanwhile, levodopa equivalent daily dose (LEDD)^28^ was calculated to evaluate the use of dopaminergic drugs. Subsequently, eligible participants underwent the baseline assessments within 7 days (same arrangement for the HCs group). In the baseline visit, all PD patients underwent neuropsychiatric examinations, including motor and non‐motor symptoms by two professional neurologists, followed by assessments of gait and fNIRS during the ON‐phase in the morning.

Specifically, all PD patients were assessed for the severity of motor symptoms by the Unified Parkinson's Disease Rating Scale section III (UPDRS‐III),^29^ for the disease stage by H&Y stage, and for the severity of gait impairments by Time Up and Go (TUG) test^30^ and Tinetti Balance and Gait.^31^ Besides, in terms of non‐motor symptoms, all PD patients were assessed for cognitive function by MMSE and Frontal Assessment Battery (FAB), for depressive symptoms by Hamilton Depression Scale‐24 (HAMD‐24), for anxiety by Hamilton Anxiety Scale (HAMA), for sleep disorders by Parkinson's disease sleep scale (PDSS) and Epworth Sleepiness Scale (ESS), and for fatigue symptoms by Fatigue Severity Scale (FSS).

**FIGURE 2:**
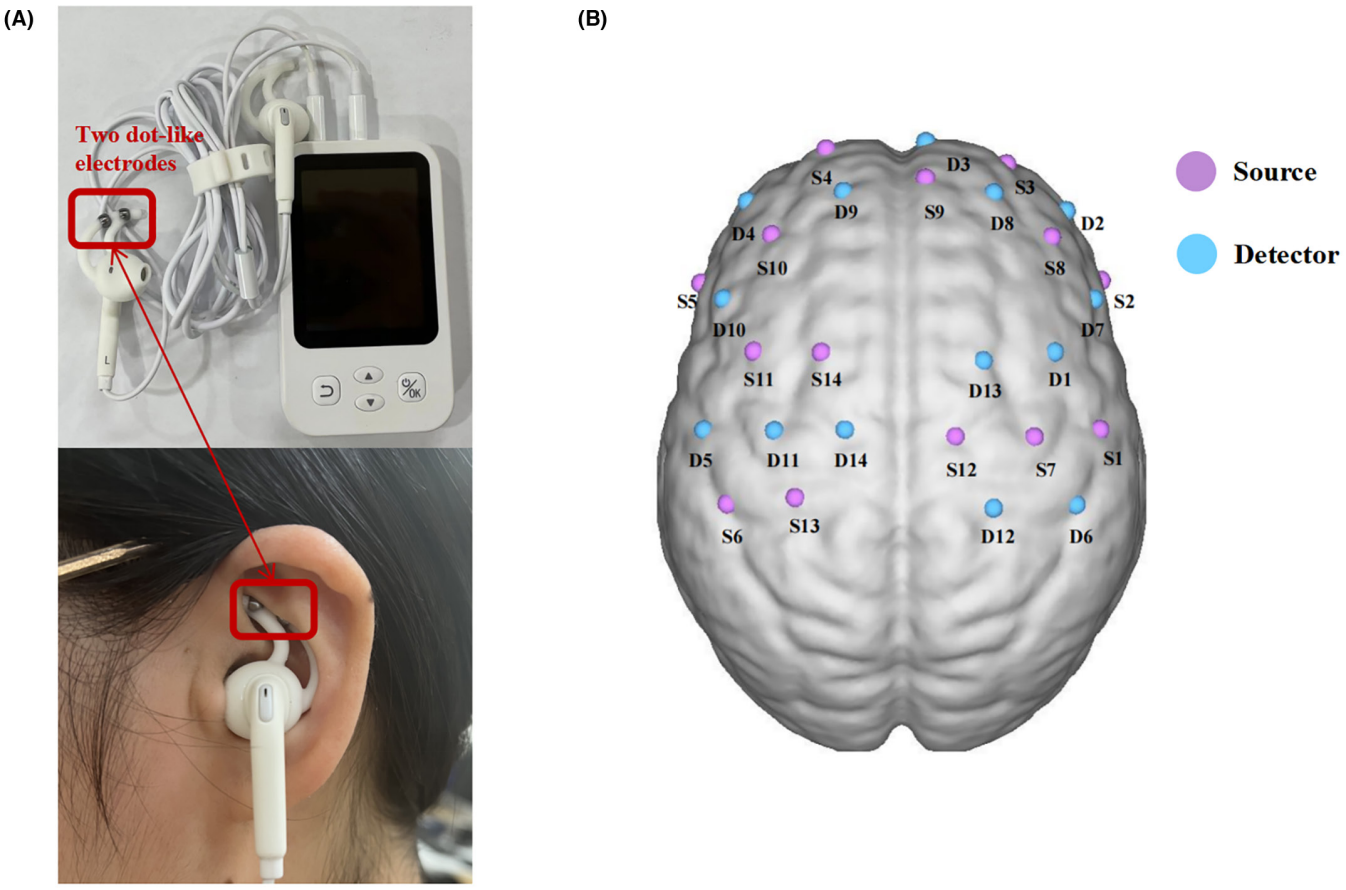
(A) Position of the taVNS stimulation (cymba conchae); (B) Schematic illustration of the fNIRS layout (35 channels, 14 sources, and 14 detectors). The nodes represent optical probes. The arrangement covers the bilateral prefrontal cortex, pre‐motor cortex, supplementary motor cortex, primary motor cortex, and primary somatosensory cortex. Details are shown in Table S1. fNIRS, functional near‐infrared spectroscopy; taVNS, transcutaneous auricular vagus nerve stimulation.

Subsequently, 26 PD patients were randomly allocated to receive active‐taVNS or sham‐taVNS stimulation in the outpatient department of Neurology (the First Affiliated Hospital of Nanjing Medical University). taVNS was conducted by transcutaneous electrical stimulation therapy instrument (tVNS501, RISHENA). Two modified dot‐like electrodes delivered the stimulation to the cymba conchae of left ear in the vicinity of the auricular branch vagus nerve (Figure 2A). Stimulation parameters: frequency = 20 Hz; pulse width = 500 μs; lasting 60 s stimulations on, alternated with 10 s off, repeat until 30 min. Every PD patient received stimulation twice daily, 30 min each time, for 7 consecutive days. The stimulation intensity was set as the maximum value the patient could tolerate without causing pain. In the sham stimulation group, the electrodes were fixed at the same position without releasing current. Out of double‐blinded purpose, all participants were informed that they might not experience any feelings from the stimulation. The second visit was scheduled for the morning after the 7‐day treatment (ON‐phase). In particular, the fNIRS and gait were preferentially accessed. In terms of clinical symptoms, only UPDRS‐III, TUG, Tinetti Balance, and Tinetti Gait were evaluated in this visit since our study mainly focused on the efficacy of gait impairment in PD patients. All participants maintained their regular medications throughout the study.

### 2.3 Gait assessment

A portable Inertial Measurement Unit (IMU) system (GYENNO Science) during a 5‐m timed TUG test was used to access gait feature. IMUs were placed on the chest, back, wrists, thighs, ankles, and insteps of the subjects via Velcro straps in order. Wirelessly transmitted mobility data were stored and analyzed by a laptop that controlled the protocols. The mean value and variability (coefficient of variation, CV) of gait feature including step length, stride velocity, stride length, arm range of motion (ROM) maximum, double support time, gait cycle, turning average duration, and turning average duration velocity were calculated (CV was calculated as standard deviation/mean × 100%).

### 2.4 Functional near infrared spectroscopy data acquisition and preprocess

A 35‐channel portable fNIRS device (Nirsmart, Huichuang) was utilized. The sample frequency was 11 Hz, and the wavelengths were 760 and 850 nm. Given that prefrontal and sensorimotor cortex activity was correlated with gait impairments in PD,^23^, ^24^, ^25^ 14 signal sources and 14 detectors placed on the bilateral prefrontal cortex (PFC), pre‐motor area (PMA), supplementary motor area (SMA), primary motor cortex (M1), and primary somatosensory cortex were chosen, which were based on the international‐used 10/20 electrode distribution system. The distribution of 35‐channels is displayed in Figure 2B, and the coordinates are shown in Table S1. A flexible headgear was used to fix the signal sources and detectors to acquire high‐precision data. Additionally, the average distance between the signal sources and detectors was set to 30 mm to contact the skin as much as possible.

The experiment conducted in a quiet room with soft light included two phases: (1) participants were instructed to stand quietly, look straight ahead, and think of nothing for 15 s and (2) when hearing the ‘start’ command, participants walked for 65 s at their comfortable pace back and forth over a 5‐m distance, with a 180‐degree turn at each end. Notably, participants were required to stand still for at least 1 min to ensure stable blood pressure before each experiment. An internal software named NirSpark (Huichuang) was used to preprocess the fNIRS data. The steps are as follows^32^: (1) convert light intensity to optical density; (2) correct motion artifacts via moving standard deviation and cubic spline interpolation method; (3) filter (0.01–0.1 Hz); and (4) convert the filtered optical density signal to oxyhemoglobin (HbO_2_) and deoxyhemoglobin (HHb) based on the modified Beer–Lambert law. Since HbO_2_ was the most used indicator to reflect alterations of cortical activity related to walking^33^, ^34^, ^35^ and showed higher sensitivity than HHb in locomotor tasks,^34^ only HbO_2_ was used in the subsequent analysis. We excluded the 5 s immediately before and after the instruction.^36^, ^37^ After that, baseline HbO_2_ was subtracted from the experimental task to assess the relative change in HbO_2_ (ΔHbO_2_) in the walking task.^36^, ^37^, ^38^ The ΔHbO_2_ was often considered a proxy for cortical activation.^37^

### 2.5 Outcomes

The primary outcome measure was the effect of taVNS on gait parameters, UPDRS‐III, TUG, Tinetti Balance, and Tinetti Gait scores. The secondary outcomes included determination of effects of taVNS on ΔHbO_2_ within the bilateral PFC, PMA, SMA, M1, and primary somatosensory cortex during the walking task.

### 2.6 Safety

Safety was evaluated by recording the number of each participant's headaches, dizziness, tinnitus, ear irritation, or skin toxicity.

### 2.7 Statistical analysis

A sample size of at least 13 patients per group was required (calculated by PASS v15 software, factorial analysis of variance using effect size, minimum power: 0.8, type I error: 0.05, numbers of factors: 2, and effect size: 0.4).

All data were analyzed using SPSS v25.0 software (IBM) and Shapiro–Wilks test was performed to assessed for normality. For baseline demographic and clinical characteristics, *χ* ^2^ test and Fisher's exact test were used for discrete variables. One‐way analysis of variance (ANOVA), Kruskal–Wallis, two‐sample *t*‐test, and Mann–Whitney test were used for continuous variables.

Later, to evaluate the severity of gait impairments in PD patients, two‐sample *t* test or Mann–Whitney test was performed on gait parameters between PD patients and HC. Besides, we compared the difference of ΔHbO_2_ between PD patients and HCs using two‐sample *t* test to verify the existing findings and provide a basis for exploring the treatment mechanism. Since the comparison of ΔHbO_2_ was performed at the channel level, we corrected the *p*‐value by false discovery rate (FDR).

Finally, to determine the effect of group and stimulation conditions on gait parameters, motor symptoms, and ΔHbO_2_, two‐way ANOVA, with group (taVNS stimulation vs. sham stimulation) and condition (pre‐stimulation vs. post‐stimulation) as the factors, was conducted. Specifically, the group main effect was assessed by comparing gait parameters, motor symptoms, and ΔHbO_2_ in the taVNS stimulation group with those in the sham stimulation group. Similarly, the stimulation condition main effect was determined using gait parameters, motor symptoms, and ΔHbO_2_ before or after taVNS stimulation, regardless of grouping. More importantly, the interactive analysis combined group and stimulation conditions. Effect sizes (*η* ^2^) were also reported. Multiple comparisons were analyzed by Bonferroni post hoc tests. Statistical significance was defined as two‐tailed *p* < 0.05.

## 3 RESULTS

### 3.1 Demographic and clinical characteristics

Twenty‐two PD patients and 14 HCs who finished the entire procedures were included in this study. Table 1 summarizes the demographic and clinical characteristics of all participants. The three groups were matched in terms of age, sex, education, MMSE scores, FAB, HAMD‐24 scores, and HAMA scores. Meanwhile, no significant differences were detected between the two subgroups of PD patients in other baseline motor and nonmotor symptoms (Table 1).

**TABLE 1:** Demographic and clinical characteristics of participants.

Characteristics	PD		HCs	*p* Value
	taVNS stimulation	Sham stimulation		
*n*	11	11	14	
Age (y)	67.64 ± 4.86	66.27 ± 6.39	63.14 ± 6.88	0.136^c^
Sex (M/F)	5/6	6/5	9/5	0.644^b^
Education (y)	12.91 ± 2.47	12.27 ± 3.13	12.21 ± 3.56	0.907^c^
MMSE	28.55 ± 1.44	28.18 ± 0.87	28.50 ± 1.16	0.693^c^
FAB	16.00 ± 1.79	16.73 ± 1.01	16.86 ± 1.17	0.466^c^
HAMA	3.18 ± 2.04	2.73 ± 1.00	2.79 ± 1.25	0.776^c^
HAMD‐24	3.64 ± 1.57	2.91 ± 1.30	2.79 ± 1.12	0.259^a^
Disease duration (y)	4.36 ± 2.50	4.05 ± 2.61	NA	0.773^d^
UPDRS‐III (ON state)	18.64 ± 10.36	14.64 ± 3.17	NA	0.244^d^
H&Y stage (ON state)	1.53 ± 0.48	1.39 ± 0.49	NA	0.519^e^
Initial side of onset of motor symptoms (R/L)	5/6	7/4	NA	0.670^f^
Tinetti balance	15.55 ± 0.69	15.36 ± 1.21	NA	0.898^e^
Tinetti gait	10.55 ± 0.93	10.82 ± 1.08	NA	0.699^e^
TUG (s)	17.03 ± 4.86	14.69 ± 3.70	NA	0.219^d^
ESS	4.73 ± 2.24	3.55 ± 4.30	NA	0.116^e^
PDSS	121.18 ± 19.76	128.64 ± 14.62	NA	0.401^e^
FSS	21.73 ± 14.62	22.36 ± 17.29	NA	0.927^d^
LEDD, mg/day	555.68 ± 220.61	556.81 ± 250.77	NA	0.991^d^

*Note*: Values are presented as the mean ± standard deviation. *p* < 0.05 was considered statistically significant.

Abbreviations: ESS, Epworth Sleepiness Scale; F, female; FAB, frontal assessment battery; FSS, Fatigue Severity Scale; H&Y stage, Hoehn and Yahr clinical rating scale; HAMA, Hamilton Anxiety Scale; HAMD‐24, Hamilton Depression Scale‐24; HCs, healthy controls; L, left; LEDD, levodopa equivalent daily dose; M, male; MMSE, Mini‐Mental State Examination; NA, not applicable; PD, Parkinson's disease; PDSS, Parkinson's Disease Sleep Scale; R, right; s, second; taVNS, transcutaneous auricular vagus nerve stimulation; TUG, Time Up and Go; UPDRS, Unified Parkinson's Disease Rating Scale; y, year.

^a^ Kruskal–Wallis.

^b^ Fisher's exact test.

^c^ One‐way ANOVA.

^d^ Two sample *t*‐test.

^e^ Mann–Whitney *U*.

^f^ Chi‐square test.

### 3.2 The difference of gait parameters between PD and HCs groups

**FIGURE 3:**
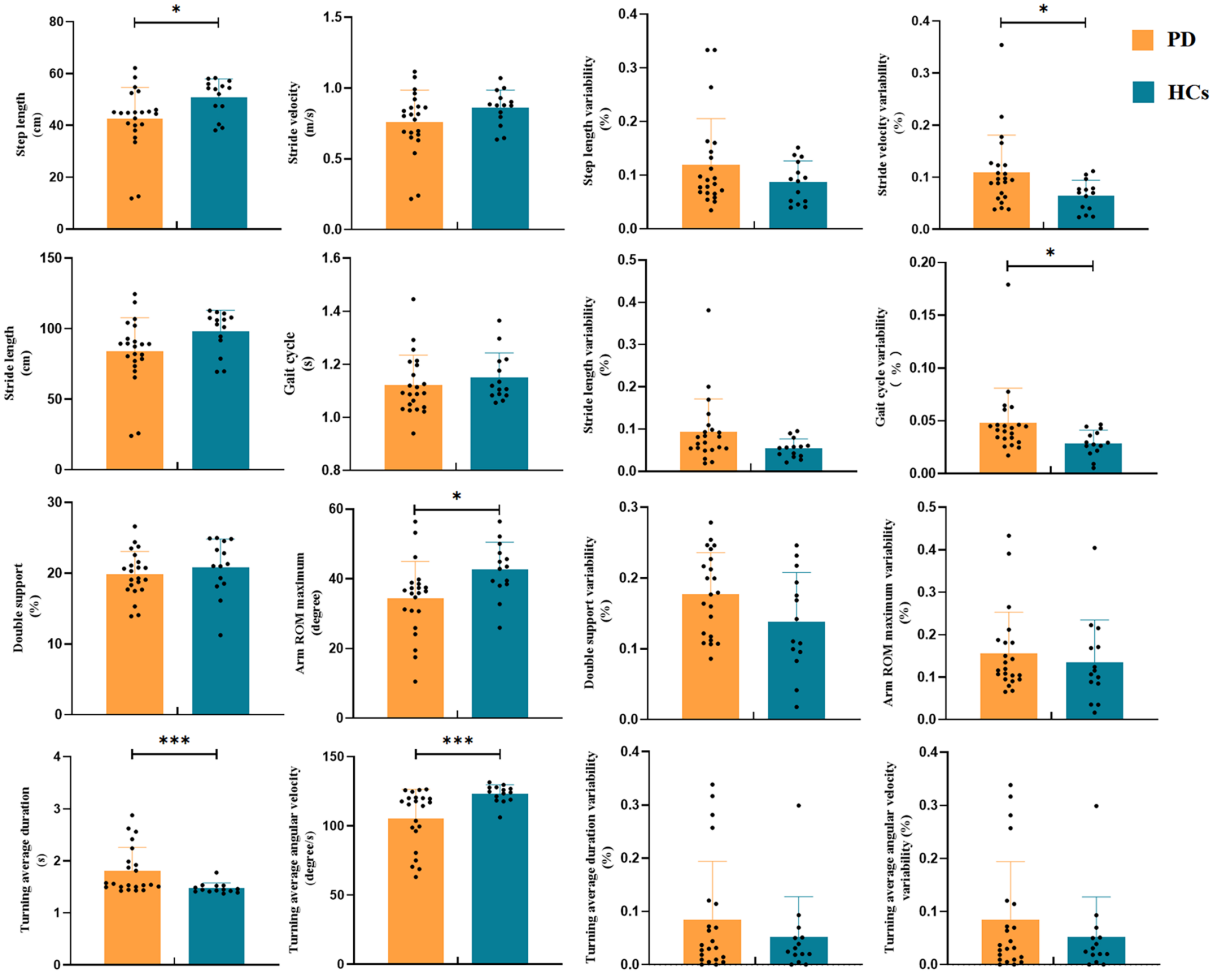
Comparing the difference of gait parameters in PD and HCs groups. The statistical threshold was set at *p* < 0.05. HCs, healthy controls; PD, Parkinson's disease. *p < 0.05, **p < 0.01, ***p < 0.001.

The inter‐group comparison showed that PD patients had decreased step length (*p* = 0.023), arm ROM maximum (*p* = 0.017), and turning average duration velocity (*p* = 0.001), while increased turning average duration (*p* = 0.001) compared with HCs group (Figure 3). Meanwhile, PD patients showed increased variability of stride velocity (*p* = 0.027) and gait cycle (*p* = 0.012) compared with HCs group (Figure 3). The remaining gait parameters showed no statistical differences between the two groups (*p* > 0.05).

### 3.3 The difference of ΔHbO_2_ between PD and HCs groups

**FIGURE 4:**
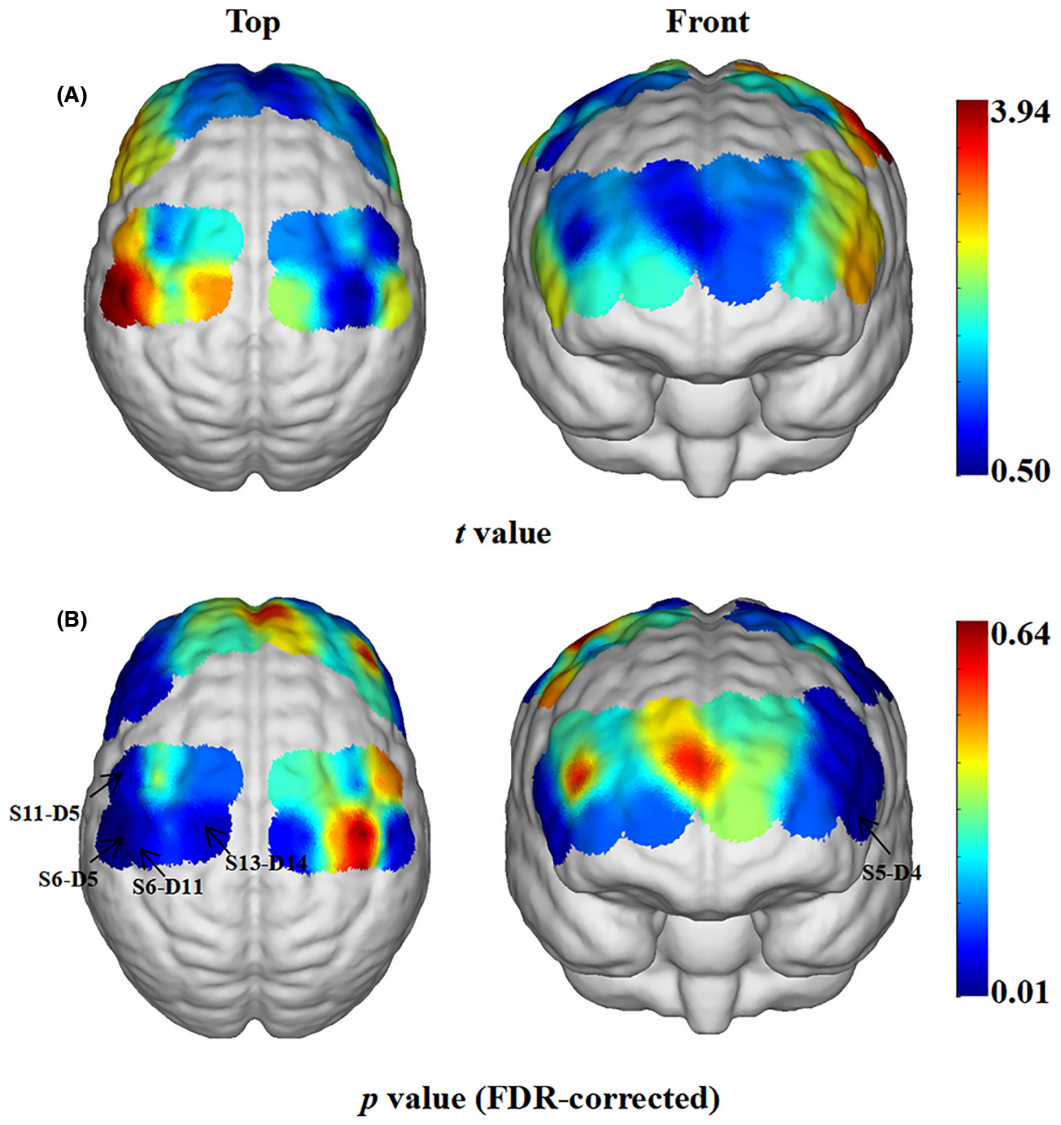
Difference of ΔHbO_2_ between PD and HCs groups. (A) *t*‐value diagram. The brain regions labeled with warm colors represent higher ΔHbO_2_ in PD than in HCs. PD patients showed higher activation in the prefrontal cortex and bilateral sensorimotor cortex than HCs. (B) *p*‐value diagram. The brain regions labeled with cold colors represent the more significant difference between groups. The ΔHbO_2_ of the left DLPFC (S5‐D4), PMA and SMA (S11‐D5), M1 (S13‐D14), and primary somatosensory cortex (S6‐D5 and S6‐D11) in the PD patients were significantly different from those in the HCs. The statistical threshold was set at *p* < 0.05 (FDR corrected). HbO_2_, oxyhemoglobin; HCs, healthy controls; FDR, false discovery rate; PD, Parkinson's disease.

PD patients showed increased ΔHbO_2_ in channel S5‐D4 (corresponding to the left dorsolateral prefrontal cortex, *p* = 0.049), S6‐D5 (corresponding to the left primary somatosensory cortex, *p* = 0.013), S6‐D11 (corresponding to the left primary somatosensory cortex, *p* = 0.049), S11‐D5 (corresponding to the left PMA and SMA, *p* = 0.049), and S13‐D14 (corresponding to the left M1, *p* = 0.049) than HCs (Figure 4). No difference was found in other channels between PD and HCs groups (*p* > 0.05).

### 3.4 Effect on gait and motor symptoms

**FIGURE 5:**
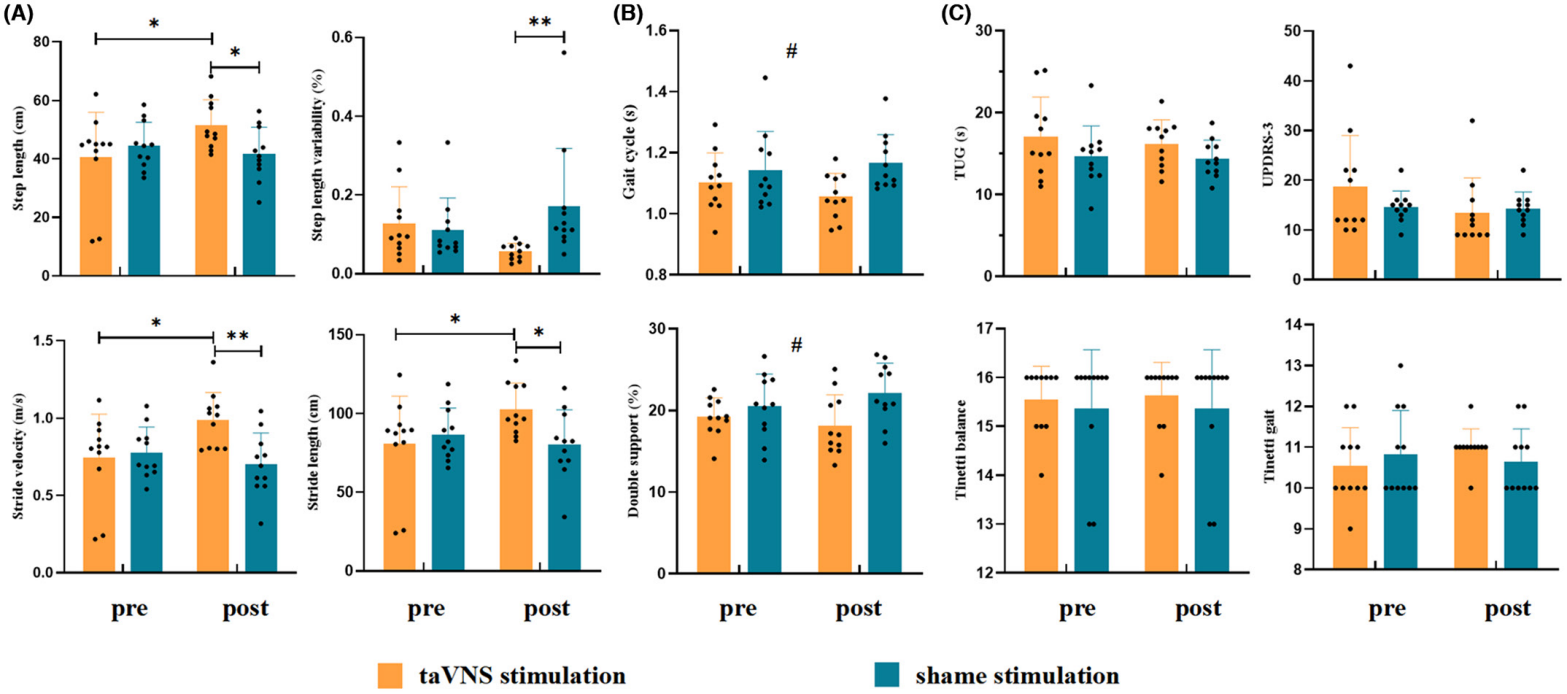
Effects of taVNS on gait parameters and motor symptoms. (A) Interaction effect on gait parameters including step length, variability of step length, stride velocity, and stride length. (B) Main effect of group (taVNS stimulation vs. sham stimulation) on gait parameters including gait cycle and double support. (C) Effect on motor symptoms including TUG, UPDRS‐3, Tinetti Balance, and Tinetti Gait scores. A Bonferroni‐corrected threshold was set at *p* < 0.05. Details are shown in Tables S2 and S3. s, seconds; taVNS, transcutaneous auricular vagus nerve stimulation; TUG, Time Up and Go; UPDRS, Unified Parkinson's Disease Rating Scale. *p < 0.05, **p < 0.01, ^#^A main effect of group.

Two‐way ANOVA showed a significant interaction between group and condition effect in gait parameters including step length (*F* = 4.506, *p* = 0.040, *η* ^2^ = 0.101), stride velocity (*F* = 6.193, *p* = 0.017, *η* ^2^ = 0.134), stride length (*F* = 4.525, *p* = 0.040, *η* ^2^ = 0.102), and step length variability (*F* = 5.226, *p* = 0.028, *η* ^2^ = 0.116) (Table S2). We also found significant group main effect in the gait cycle (*F* = 6.055, *p* = 0.018, *η* ^2^ = 0.131) and double support (*F* = 6.388, *p* = 0.016, *η* ^2^ = 0.138) (Table S2). No difference was found in the stimulation condition main effect (*p* > 0.05, Table S2). For interaction effect, Bonferroni post hoc tests revealed that, in the taVNS stimulation group, PD patients in the post‐stimulation condition presented significantly increased step length (*p* = 0.022), stride length (*p* = 0.025), and stride velocity (*p* = 0.011) compared with those in the pre‐stimulation (Figure 5A). Moreover, in the post‐stimulation condition, PD patients in the taVNS stimulation group exhibited increased step length (*p* = 0.035), stride length (*p* = 0.022), and stride velocity (*p* = 0.003) and decreased step length variability (*p* = 0.008) than those in the sham stimulation group (Figure 5A). For group main effect, Bonferroni post hoc tests showed that PD patients in the taVNS stimulation group showed decreased gait cycle (*p* = 0.018) and double support (*p* = 0.016) than those in the sham stimulation group (Figure 5B).

No difference was found in the group main effect, condition main effect, or interaction when analyzing the motor symptoms including UPDRS‐III, TUG, Tinetti Balance, and Gait scores (*p* > 0.05, Figure 5C).

### 3.5 Effect on ΔHbO_2_

**FIGURE 6:**
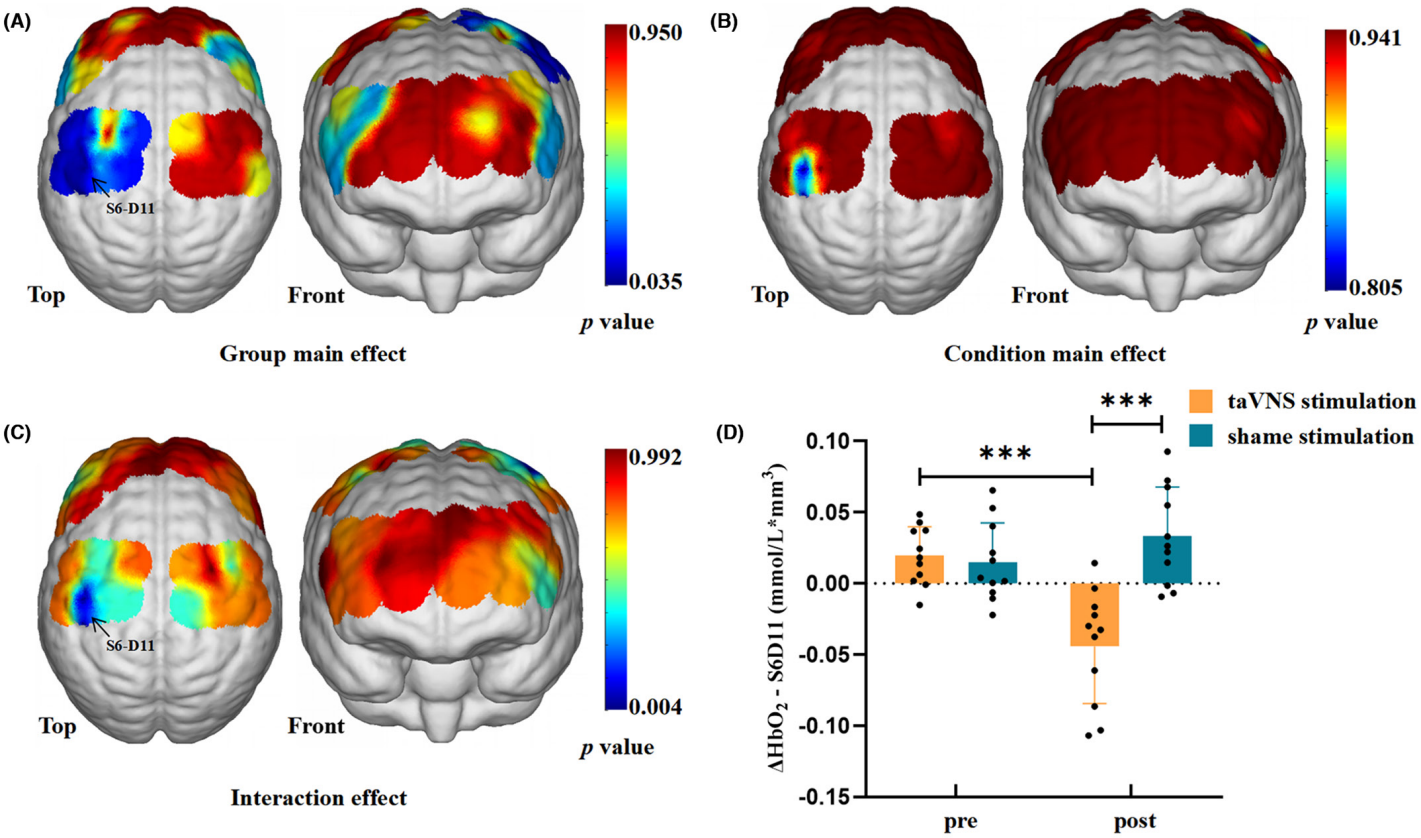
(A) Main effect of group (taVNS stimulation vs. sham stimulation). Significant differences obtained from the main effect of group were in the S6‐D11 (corresponding to the left primary somatosensory cortex). (B) Main effect of stimulation condition (pre‐stimulation vs. post‐stimulation). No significant differences were obtained from the main effect of condition. (C) Interaction between group and condition effect. Interaction between group and stimulation effect was found in the S6‐D11 (corresponding to the left primary somatosensory cortex). The color bar indicates *p* values from two‐way ANOVA, with group (taVNS stimulation vs. sham stimulation) and condition (pre‐stimulation vs. post‐stimulation) as the factors. The statistical threshold was set at *p* < 0.05 (FDR corrected). (D) Post‐hoc tests in the S6‐D11. A Bonferroni‐corrected threshold was set at *p* < 0.05 for multiple comparison. Error bars indicate standard deviations. ANOVA, analyses of variance; D, detector; FDR, false discovery rate; HbO_2_, oxyhemoglobin; S, source; taVNS, transcutaneous auricular vagus nerve stimulation. ***p < 0.001.

Two‐way ANOVA showed a significant group main effect for ΔHbO_2_ in channel S6‐D11 corresponding to the left primary somatosensory cortex (*F* = 14.632, *p* = 0.035, *η* ^2^ = 0.268) (Figure 6A). Furthermore, a significant interaction between group and condition effect was also detected in channel S6‐D11 (*F* = 18.567, *p* = 0.004, *η* ^2^ = 0.317) (Figure 6C). No difference was found in condition main effect (*p* > 0.05, Figure 6B). Bonferroni post hoc tests revealed that, in the taVNS stimulation group, the ΔHbO_2_ in channel S6‐D11 of PD patients in the post‐stimulation condition was significantly decreased compared with that in the pre‐stimulation condition (*p* < 0.001) (Figure 6D). In addition, in the post‐stimulation condition, the relative HbO_2_ in channel S6‐D11 of PD patients in the taVNS stimulation group was lower than that in the sham stimulation group (*p* < 0.001) (Figure 6D).

### 3.6 Safety

taVNS possessed good tolerance since there were no reports of adverse events caused by stimulation.

## 4 DISCUSSION

This study investigated the effect of taVNS on gait impairments and brain activity in PD. First, PD patients had decreased step length, arm ROM maximum, and turning average duration velocity, while increased turning average duration, stride velocity variability, and gait cycle relative to the HCs group. Simultaneously, improvements in gait characteristics, including step length, stride velocity, stride length, step length variability, gait cycle, and double support after 7‐day taVNS therapy, were significant. Second, PD patients had higher ΔHbO_2_ in the left dorsolateral prefrontal cortex (DLPFC), PMA, SMA, M1, and primary somatosensory cortex than controls during usual walking. Furthermore, we found that hemodynamic responses of primary somatosensory cortex in PD patients during usual walking were significantly decreased by taVNS, which might be related to the improvement of gait impairments.

Accumulating literature uncovered that PD patients showed impaired pace, step length, and rhythmicity compared with age‐matched healthy adults.^2^ Consistent with these findings, the differences in gait characteristics between PD and HCs groups in our study reflected the phenomenon of enhanced volitional control and increased gait instability in PD patients during usual walking.^2^ Studies in rats uncovered that VNS therapy could improve locomotion or locomotor asymmetry related to PD.^6^ Meanwhile, cervical nVNS^11^, ^13^ and single taVNS^14^ therapy was found to ameliorate gait in PD patients. The IAMZs electrical stimulation was found to have a short‐term effect on improving gait^16^ of PD patients. Similarly, significant increases in velocity, step length, and stride length with taVNS therapy were detected in our study compared with baseline, indicating that PD patients were walking at a faster pace after taVNS therapy. Additionally, a significant reduction in the step length variability with taVNS therapy was observed relative to patients receiving sham stimulation, revealing that gait stability in PD patients was improved. Considering that the step length variability was a dopa‐resistant gait characteristic,^39^ we surmised that dopa‐resistant gait impairments could benefit from taVNS therapy, which was in accordance with a previous study.^12^ In terms of other outcomes, Mondal et al.^13^ found that cervical nVNS therapy caused a significant improvement in the UPDRS‐III scores in PD patients with FOG compared with that after sham stimulation. Cakmak et al.^15^ also found that IAMZs electrical stimulation improved UPDRS motor scores in the short term. However, we found no statistical difference in the UPDRS‐III, TUG, Tinetti Gait, or Balance scores. Explanations for this phenomenon are as follows: first, UPDRS‐III scores in our study showed a downward trend after taVNS therapy. Nevertheless, treatment time, stimulation sites, and stimulation parameters were inconsistent with those in the study mentioned earlier. Especially, IAMZs could stimulate not only the vagus nerve but also the facial nerve branches, the trigeminal nerve, the C2 spinal nerve, and sympathetic nerves.^15^ The difference between the neural networks related to auricular vagus nerve and the IAMZs might also contribute to the diversity above. Second, a more robust statistical methodology was conducted in our study which might be a significant contributor to this discrepancy.

Cerebral gait control is mainly accomplished through direct and indirect pathways: the direct pathway from the M1 to the central pattern generators of the spinal cord participates in the automatic control of gait, and the indirect pathway from the PFC and PMA to the basal ganglia, and then to the brainstem motor center can regulate gait based on challenging situations.^24^, ^40^ In our study, the left DLPFC and sensorimotor cortex in PD patients without cognitive impairment were significantly activated during walking compared with HCs, indicating that abnormalities occurred in both direct and indirect gait control pathways might induce gait impairments in PD patients. Consistently, a fNIRS study observed abnormal activation in the PFC and M1, accompanied by decreased Soleus H‐reflex in PD patients relative to controls, which suggested that gait instability in PD might partly arise from the abnormal sensorimotor function that reduced the sensitivity of peripheral reflexes.^24^ The primary somatosensory cortex is essential for locomotor control since it encodes sensory inputs and generates a motor response.^41^ Evidence revealed a cortical–cortical (primary somatosensory cortex‐M1) loop to ensure sensorimotor integration function.^42^ Moreover, a mice study identified a direct primary somatosensory cortex‐spinal cord pathway that could regulate the lumbar motor network independently of the motor cortex and other supraspinal motor centers.^41^ Further, impaired proprioception has been shown to contribute to PD.^43^ Hence, we speculated that PD patients had abnormal sensorimotor integration and compensated by enhancing the activation in the primary somatosensory cortex. Interestingly, the interactive analysis uncovered that the activation in the primary somatosensory cortex decreased in PD patients when walking compared with the pre‐stimulation baseline. Concurrently, in the post‐stimulation state, the activation in the primary somatosensory cortex in the taVNS active stimulation group was significantly lower than that in the sham stimulation group. Considering that taVNS could induce widespread, diffuse cortical and subcortical effects,^18^, ^19^ we conjectured that taVNS remodeled sensorimotor integration function in PD patients so that compensatory activation in the primary somatosensory cortex was non‐essential after taVNS treatment. Overall, our results suggested that taVNS therapy could improve gait disturbance in PD patients, which might be related to a neural mechanism.

Apart from small sample size, some limitation should be considered. First, limited detection depth of fNIRS system hindered the detection of activation in subcortical structures. However, the portable fNIRS enabled us to capture functional alterations in the subjects' cerebral cortex during actual walking, which was beneficial for us to understand the neural mechanism of gait impairments in PD better. Second, dopaminergic therapies might modulate cortical function,^44^ yet our fNIRS examinations were performed after dopaminergic drug intake. Nevertheless, LEDD was matched in the active‐ and sham‐stimulation group, which had reduced the influence brought by the drug as much as possible. Third, all participants were assessed for cognitive function by MMSE and FAB which showed no difference among the three groups (taVNS stimulation vs. sham stimulation vs. HC group). However, Montreal Cognitive Assessment as a better marker for cognitive decline in PD population should be evaluated. Fourth, the electrodes were fixed at the same position without releasing current in the sham stimulation group^17^ in our study. Although this sham stimulation group could eliminate the interference of placebo, this was not the best sham‐stimulation method. Considering that transcutaneous electrical stimulation of the left earlobe is a commonly used sham‐stimulation method in taVNS research,^18^, ^45^ our result should be replicated with a real sham stimulation in the follow‐up study. Future studies should increase the sample size and use simultaneous NIRS‐functional magnetic resonance imaging to replicate and further probe the efficacy and potential mechanism of taVNS therapy in PD gait impairments.

## 5 CONCLUSION

Our findings suggested that taVNS could relieve gait impairments and remodel sensorimotor integration in PD patients. The results provided insights into the neural mechanism of taVNS and a new neuromodulation method for treating gait impairments in PD patients.

## AUTHOR CONTRIBUTIONS

Heng Zhang: Conceptualization, Data acquisition, Formal analysis, interpretation, Writing‐original draft, Writing‐review & editing. Xing‐yue Cao: Conceptualization, Data acquisition, Writing‐review & editing. Li‐na Wang, Qing Tong, Hui‐min Sun, and Cai‐ting Gan: Data acquisition, Writing‐review & editing. Ai‐di Shan: Grouping and intervention of participants. Yong‐sheng Yuan: Conceptualization, Data acquisition, Writing‐review & editing, Funding acquisition. Ke‐zhong Zhang: Conceptualization, Data acquisition, safety assessment, Writing‐review & editing, Study supervision, Funding acquisition.

## FUNDING INFORMATION

This work was funded by the National Natural Science Foundation of China (82271273) and the Jiangsu Social Development Project (BE2022808).

## CONFLICT OF INTEREST STATEMENT

Authors declare no conflict of interest.

## CONSENT

Written informed consent for publication was obtained from all participants.

## Supporting information


Tables S1–S3

## Data Availability

The original contributions presented in the study are included in the article/Supporting Information, further inquiries can be directed to the corresponding author/s on reasonable request.

## References

[1] Bloem BR , Okun MS , Klein C . Parkinson's disease. Lancet. 2021;397(10291):2284‐2303. 33848468 10.1016/S0140-6736(21)00218-X

[2] Mirelman A , Bonato P , Camicioli R , et al. Gait impairments in Parkinson's disease. Lancet Neurol. 2019;18(7):697‐708. 30975519 10.1016/S1474-4422(19)30044-4

[3] Curtze C , Nutt JG , Carlson‐Kuhta P , Mancini M , Horak FB . Levodopa is a double‐edged sword for balance and gait in people with Parkinson's disease. Mov Disord. 2015;30(10):1361‐1370. 26095928 10.1002/mds.26269 PMC4755510

[4] DeGiorgio CM , Schachter SC , Handforth A , et al. Prospective long‐term study of vagus nerve stimulation for the treatment of refractory seizures. Epilepsia. 2000;41(9):1195‐1200. 10999559 10.1111/j.1528-1157.2000.tb00325.x

[5] Marangell LB , Martinez JM , Niazi SK . Vagus nerve stimulation as a potential option for treatment‐resistant depression. Clin Neurosci Res. 2004;4(1–2):89‐94.

[6] Farrand AQ , Helke KL , Gregory RA , Gooz M , Hinson VK , Boger HA . Vagus nerve stimulation improves locomotion and neuronal populations in a model of Parkinson's disease. Brain Stimul. 2017;10(6):1045‐1054. 28918943 10.1016/j.brs.2017.08.008 PMC5675746

[7] Jiang Y , Cao Z , Ma H , et al. Auricular vagus nerve stimulation exerts antiinflammatory effects and immune regulatory function in a 6‐OHDA model of Parkinson's disease. Neurochem Res. 2018;43(11):2155‐2164. 30311182 10.1007/s11064-018-2639-z

[8] Farrand AQ , Helke KL , Aponte‐Cofresi L , et al. Effects of vagus nerve stimulation are mediated in part by TrkB in a Parkinson's disease model. Behav Brain Res. 2019;373:112080. 31301412 10.1016/j.bbr.2019.112080 PMC6701465

[9] Farrand AQ , Verner RS , McGuire RM , Helke KL , Hinson VK , Boger HA . Differential effects of vagus nerve stimulation paradigms guide clinical development for Parkinson's disease. Brain Stimul. 2020;13(5):1323‐1332. 32629028 10.1016/j.brs.2020.06.078

[10] Kin I , Sasaki T , Yasuhara T , et al. Vagus nerve stimulation with mild stimulation intensity exerts anti‐inflammatory and neuroprotective effects in Parkinson's disease model rats. Biomedicine. 2021;9(7):789. 10.3390/biomedicines9070789 PMC8301489 34356853

[11] Mondal B , Choudhury S , Simon B , Baker MR , Kumar H . Noninvasive vagus nerve stimulation improves gait and reduces freezing of gait in Parkinson's disease. Mov Disord. 2019;34(6):917‐918. 30869809 10.1002/mds.27662

[12] Morris R , Yarnall AJ , Hunter H , Taylor JP , Baker MR , Rochester L . Noninvasive vagus nerve stimulation to target gait impairment in Parkinson's disease. Mov Disord. 2019;34(6):918‐919. 30889295 10.1002/mds.27664

[13] Mondal B , Choudhury S , Banerjee R , et al. Non‐invasive vagus nerve stimulation improves clinical and molecular biomarkers of Parkinson's disease in patients with freezing of gait. NPJ Parkinsons Dis. 2021;7(1):46. 34045464 10.1038/s41531-021-00190-x PMC8160211

[14] Marano M , Anzini G , Musumeci G , et al. Transcutaneous auricular vagus stimulation improves gait and reaction time in Parkinson's disease. Mov Disord. 2022;37:2163‐2164. 35861362 10.1002/mds.29166 PMC9796229

[15] Cakmak YO , Apaydin H , Kiziltan G , et al. Rapid alleviation of Parkinson's disease symptoms via electrostimulation of intrinsic auricular muscle zones. Front Hum Neurosci. 2017;11:338. 28701941 10.3389/fnhum.2017.00338 PMC5487461

[16] Cakmak YO , Ozsoy B , Ertan S , et al. Intrinsic auricular muscle zone stimulation improves walking parameters of Parkinson's patients faster than levodopa in the motion capture analysis: a pilot study. Front Neurol. 2020;11:546123. 33117256 10.3389/fneur.2020.546123 PMC7575762

[17] Wu DD , Ma JX , Zhang LP , Wang SR , Tan BT , Jia GW . Effect and safety of transcutaneous auricular vagus nerve stimulation on recovery of upper limb motor function in subacute ischemic stroke patients: a randomized pilot study. Neural Plast. 2020;2020:8841752. 32802039 10.1155/2020/8841752 PMC7416299

[18] Badran BW , Dowdle LT , Mithoefer OJ , et al. Neurophysiologic effects of transcutaneous auricular vagus nerve stimulation (taVNS) via electrical stimulation of the tragus: a concurrent taVNS/fMRI study and review. Brain Stimul. 2018;11(3):492‐500. 29361441 10.1016/j.brs.2017.12.009 PMC6487660

[19] Engineer ND , Riley JR , Seale JD , et al. Reversing pathological neural activity using targeted plasticity. Nature. 2011;470(7332):101‐114. 21228773 10.1038/nature09656 PMC3295231

[20] Steinbrink J , Villringer A , Kempf F , Haux D , Boden S , Obrig H . Illuminating the BOLD signal: combined fMRI‐fNIRS studies. Magn Reson Imaging. 2006;24(4):495‐505. 16677956 10.1016/j.mri.2005.12.034

[21] Bishnoi A , Holtzer R , Hernandez ME . Brain activation changes while walking in adults with and without neurological disease: systematic review and meta‐analysis of functional near‐infrared spectroscopy studies. Brain Sci. 2021;11(3):291. 33652706 10.3390/brainsci11030291 PMC7996848

[22] Menant JC , Maidan I , Alcock L , et al. A consensus guide to using functional near‐infrared spectroscopy in posture and gait research. Gait Posture. 2020;82:254‐265. 32987345 10.1016/j.gaitpost.2020.09.012

[23] Stuart S , Vitorio R , Morris R , Martini DN , Fino PC , Mancini M . Cortical activity during walking and balance tasks in older adults and in people with Parkinson's disease: a structured review. Maturitas. 2018;113:53‐72. 29903649 10.1016/j.maturitas.2018.04.011 PMC6448561

[24] Al‐Yahya E , Mahmoud W , Daan M , Esser P , Dawes H . Neural substrates of cognitive motor interference during walking; peripheral and central mechanisms. Front Hum Neurosci. 2019;12:536. 30687049 10.3389/fnhum.2018.00536 PMC6333849

[25] Ranchet M , Hoang I , Cheminon M , et al. Changes in prefrontal cortical activity during walking and cognitive functions among patients with Parkinson's disease. Front Neurol. 2020;11:601686. 33362703 10.3389/fneur.2020.601686 PMC7758480

[26] Postuma RB , Berg D , Stern M , et al. MDS clinical diagnostic criteria for Parkinson's disease. Mov Disord. 2015;30(12):1591‐1601. 26474316 10.1002/mds.26424

[27] Hoehn MM , Yahr MD . Parkinsonism: onset, progression and mortality. Neurology. 1967;17(5):427‐442. 6067254 10.1212/wnl.17.5.427

[28] Tomlinson CL , Stowe R , Patel S , Rick C , Gray R , Clarke CE . Systematic review of levodopa dose equivalency reporting in Parkinson's disease. Mov Disord. 2010;25(15):2649‐2653. 21069833 10.1002/mds.23429

[29] Movement Disorder Society Task Force on Rating Scales for Parkinson's D . The Unified Parkinson's Disease Rating Scale (UPDRS): status and recommendations. Mov Disord. 2003;18(7):738‐750. 12815652 10.1002/mds.10473

[30] Morris S , Morris ME , Iansek R . Reliability of measurements obtained with the timed “up & go” test in people with Parkinson disease. Phys Ther. 2001;81(2):810‐818. 11175678 10.1093/ptj/81.2.810

[31] Tinetti ME . Performance‐oriented assessment of mobility problems in elderly patients. J Am Geriatr Soc. 1986;34(2):119‐126. 3944402 10.1111/j.1532-5415.1986.tb05480.x

[32] Tan HX , Wei QC , Chen Y , et al. The immediate effects of intermittent theta burst stimulation of the cerebellar vermis on cerebral cortical excitability during a balance task in healthy individuals: a pilot study. Front Hum Neurosci. 2021;15:748241. 34867241 10.3389/fnhum.2021.748241 PMC8632863

[33] Harada T , Miyai I , Suzuki M , Kubota K . Gait capacity affects cortical activation patterns related to speed control in the elderly. Exp Brain Res. 2009;193(3):445‐454. 19030850 10.1007/s00221-008-1643-y

[34] Miyai I , Tanabe HC , Sase I , et al. Cortical mapping of gait in humans: a near‐infrared spectroscopic topography study. Neuroimage. 2001;14(5):1186‐1192. 11697950 10.1006/nimg.2001.0905

[35] Suzuki M , Miyai I , Ono T , et al. Prefrontal and premotor cortices are involved in adapting walking and running speed on the treadmill: an optical imaging study. Neuroimage. 2004;23(3):1020‐1026. 15528102 10.1016/j.neuroimage.2004.07.002

[36] Orcioli‐Silva D , Vitorio R , Nobrega‐Sousa P , et al. Levodopa facilitates prefrontal cortex activation during dual task walking in Parkinson disease. Neurorehabil Neural Repair. 2020;34(7):589‐599. 32449460 10.1177/1545968320924430

[37] Orcioli‐Silva D , Vitorio R , Beretta VS , et al. Is cortical activation during walking different between Parkinson's disease motor subtypes? J Gerontol A Biol Sci Med Sci. 2021;76(4):561‐567. 32674140 10.1093/gerona/glaa174

[38] Maidan I , Nieuwhof F , Bernad‐Elazari H , et al. The role of the frontal lobe in complex walking among patients with Parkinson's disease and healthy older adults: an fNIRS study. Neurorehabil Neural Repair. 2016;30(10):963‐971. 27221042 10.1177/1545968316650426

[39] Rochester L , Galna B , Lord S , et al. Decrease in Abeta42 predicts dopa‐resistant gait progression in early Parkinson disease. Neurology. 2017;88(16):1501‐1511. 28330963 10.1212/WNL.0000000000003840 PMC5395075

[40] Bohnen NI , Jahn K . Imaging: what can it tell us about Parkinsonian gait? Mov Disord. 2013;28(11):1492‐1500. 24132837 10.1002/mds.25534 PMC3801220

[41] Karadimas SK , Satkunendrarajah K , Laliberte AM , et al. Sensory cortical control of movement. Nat Neurosci. 2020;23(1):75‐84. 31740813 10.1038/s41593-019-0536-7

[42] Hooks BM . Sensorimotor convergence in circuitry of the motor cortex. Neuroscientist. 2017;23(3):251‐263. 27091827 10.1177/1073858416645088

[43] Abbruzzese G , Berardelli A . Sensorimotor integration in movement disorders. Mov Disord. 2003;18(3):231‐240. 12621626 10.1002/mds.10327

[44] Orcioli‐Silva D , Vitorio R , Nobrega‐Sousa P , et al. Cortical activity underlying gait improvements achieved with dopaminergic medication during usual walking and obstacle avoidance in Parkinson disease. Neurorehabil Neural Repair. 2021;35(5):406‐418. 33754884 10.1177/15459683211000736

[45] Borgmann D , Rigoux L , Kuzmanovic B , et al. Technical note: modulation of fMRI brainstem responses by transcutaneous vagus nerve stimulation. Neuroimage. 2021;244:118566. 34509623 10.1016/j.neuroimage.2021.118566

