# Supplementary material for: Transcutaneous auricular vagus nerve stimulation improves gait and cortical activity in Parkinson's disease: A pilot randomized study
Source: CNS Neurosci Ther. 2023 Jun 13;29(12):3889–900. doi: 10.1111/cns.14309 (PMC10651956; doi:10.1111/cns.14309)
Supplement: Supplementary file 1 — Tables S1–S3 [file CNS-29-3889-s001.doc]

**Table 1. The MNI coordinates and Brodmann areas corresponding to the measurement channels.**

| Channel | MNI coordinates | | | Brodmann areas (Talairach daemon) |
| --- | --- | --- | --- | --- |
|  | x | y | z |  |
| CH1 (S1-D1) | 52 | -2 | 55 | 6: Pre-Motor and Supplementary Motor Cortex |
| CH2 (S1-D6) | 56 | -24 | 57 | 2: Primary Somatosensory Cortex |
| CH3 (S2-D2) | 55 | 42 | 2 | 47: Dorsolateral Prefrontal Cortex |
| CH4 (S2-D7) | 60 | 29 | 14 | 45: Dorsolateral Prefrontal Cortex |
| CH5 (S3-D2) | 42 | 61 | 5 | 10: Frontopolar Area |
| CH6 (S3-D3) | 20 | 72 | 8 | 10: Frontopolar Area |
| CH7 (S3-D8) | 29 | 63 | 20 | 10: Frontopolar Area |
| CH8 (S4-D3) | -11 | 73 | 10 | 10: Frontopolar Area |
| CH9 (S4-D4) | -35 | 62 | 11 | 10: Frontopolar Area |
| CH10 (S4-D9) | -21 | 66 | 25 | 10: Frontopolar Area |
| CH11 (S5-D4) | -52 | 43 | 10 | 10: Dorsolateral Prefrontal Cortex |
| CH12 (S5-D10) | -56 | 29 | 24 | 10: Dorsolateral Prefrontal Cortex |
| CH13 (S6-D5) | -56 | -23 | 57 | 2: Primary Somatosensory Cortex |
| CH14 (S6-D11) | -45 | -23 | 67 | 3: Primary Somatosensory Cortex |
| CH15 (S7-D1) | 42 | -2 | 63 | 6: Pre-Motor and Supplementary Motor Cortex |
| CH16 (S7-D6) | 45 | -23 | 67 | 2: Primary Somatosensory Cortex |
| CH17 (S7-D12) | 34 | -24 | 73 | 4: Primary Motor Cortex |
| CH18 (S7-D13) | 31 | -5 | 69 | 6: Pre-Motor and Supplementary Motor Cortex |
| CH19 (S8-D2) | 47 | 49 | 18 | 10: Frontopolar Area |
| CH20 (S8-D7) | 52 | 36 | 29 | 46: Dorsolateral Prefrontal Cortex |
| CH21 (S8-D8) | 36 | 51 | 31 | 10: Frontopolar Area |
| CH22 (S9-D3) | 7 | 69 | 24 | 10: Frontopolar Area |
| CH23 (S9-D8) | 16 | 60 | 34 | 9: Dorsolateral Prefrontal Cortex |
| CH24 (S9-D9) | -8 | 61 | 37 | 9: Dorsolateral Prefrontal Cortex |
| CH25 (S10-D4) | -43 | 51 | 24 | 10: Frontopolar Area |
| CH26 (S10-D9) | -29 | 53 | 37 | 9: Dorsolateral Prefrontal Cortex |
| CH27 (S10-D10) | -46 | 35 | 38 | 9: Dorsolateral Prefrontal Cortex |
| CH28 (S11-D5) | -51 | -2 | 56 | 6: Pre-Motor and Supplementary Motor Cortex |
| CH29 (S11-D11) | -40 | -1 | 64 | 6: Pre-Motor and Supplementary Motor Cortex |
| CH30 (S12-D12) | 21 | -25 | 77 | 4: Primary Motor Cortex |
| CH31 (S12-D13) | 20 | -3 | 77 | 6: Pre-Motor and Supplementary Motor Cortex |
| CH32(S13-D11) | -35 | -22 | 74 | 4: Primary Motor Cortex |
| CH33 (S13-D14) | -23 | -21 | 77 | 4: Primary Motor Cortex |
| CH34(S14-D11) | -31 | -1 | 69 | 6: Pre-Motor and Supplementary Motor Cortex |
| CH35(S14-D14) | -19 | 0 | 76 | 6: Pre-Motor and Supplementary Motor Cortex |

*MNI* Montreal Neurological Institute, *CH* Channel, *S* Source, *D* Decteor

**Table 2. Results of two-ways ANOVA for gait parameters.**

| **Gait parameters** |  | **taVNS stimulation**  **（mean±SD）** | **sham stimulation**  **（mean±SD）** | **Group**  **(taVNS stimulation vs sham stimulation)** | | | **Condition**  **(pre- stimulation vs post- stimulation)** | | | **Interaction**  **(group × condition)** | | |
| --- | --- | --- | --- | --- | --- | --- | --- | --- | --- | --- | --- | --- |
|  |  | | | ***F* (1,20)** | ***p*** | ***η*^2^** | ***F* (1,20)** | ***p*** | ***η*^2^** | ***F* (1,20)** | ***p*** | ***η*^2^** |
| Step length  (cm) | Before | 40.70±15.25 | 44.46±8.09 | 0.914 | 0.345 | 0.022 | 1.587 | 0.215 | 0.038 | 4.506 | **0.040**^*^ | 0.101 |
|  | After | 51.59±8.62 | 41.68±9.15 |  |  |  |  |  |  |  |  |  |
| Stride velocity  (m/s) | Before | 0.75±0.28 | 0.78±0.16 | 4.023 | 0.052 | 0.091 | 1.679 | 0.202 | 0.040 | 6.193 | **0.017**^*^ | 0.134 |
|  | After | 0.99±0.18 | 0.70±0.20 |  |  |  |  |  |  |  |  |  |
| Stride length  (cm) | Before | 80.81±30.16 | 86.78±16.69 | 1.513 | 0.226 | 0.036 | 1.355 | 0.251 | 0.033 | 4.525 | **0.040**^*^ | 0.102 |
|  | After | 102.72±16.66 | 80.37±21.98 |  |  |  |  |  |  |  |  |  |
| Gait cycle  (s) | Before | 1.10±0.10 | 1.14±0.13 | 6.055 | 0.018 | 0.131 | 0.119 | 0.732 | 0.003 | 1.383 | 0.247 | 0.033 |
|  | After | 1.06±0.07 | 1.17±0.09 |  |  |  |  |  |  |  |  |  |
| Double support  (%) | Before | 19.22±2.33 | 20.49±3.94 | 6.388 | 0.016 | 0.138 | 0.078 | 0.781 | 0.002 | 1.712 | 0.198 | 0.041 |
|  | After | 18.14±3.79 | 22.16±3.61 |  |  |  |  |  |  |  |  |  |
| Arm ROM maximum (degree) | Before | 34.43±9.60 | 34.23±12.07 | 1.010 | 0.321 | 0.025 | 1.446 | 0.236 | 0.035 | 0.881 | 0.354 | 0.022 |
|  | After | 40.66±10.28 | 35.00±5.44 |  |  |  |  |  |  |  |  |  |
| Turning average duration (s) | Before | 1.84±0.50 | 1.77±0.41 | 0.035 | 0.852 | 0.001 | 0.920 | 0.343 | 0.022 | 0.161 | 0.690 | 0.004 |
|  | After | 1.67±0.39 | 1.70±0.35 |  |  |  |  |  |  |  |  |  |
| Turing average angular velocity (degree/s) | Before | 104.28±23.18 | 106.68±19.42 | 0.005 | 0.942 | 0.000 | 1.219 | 0.276 | 0.030 | 0.110 | 0.742 | 0.003 |
|  | After | 112.81±18.53 | 111.27±17.17 |  |  |  |  |  |  |  |  |  |
| **CV** |  | | | | | | | | | | | |
| Step length variability (%) | Before | 0.13±0.09 | 0.11±0.08 | 2.872 | 0.098 | 0.067 | 0.038 | 0.846 | 0.001 | 5.226 | **0.028**^*^ | 0.116 |
|  | After | 0.06±0.02 | 0.17±0.15 |  |  |  |  |  |  |  |  |  |
| Stride velocity variability (%) | Before | 0.11±0.06 | 0.11±0.09 | 1.789 | 0.189 | 0.043 | 0.634 | 0.431 | 0.016 | 2.599 | 0.115 | 0.061 |
|  | After | 0.07±0.02 | 0.12±0.07 |  |  |  |  |  |  |  |  |  |
| Stride length variability (%) | Before | 0.09±0.06 | 0.10±0.10 | 2.176 | 0.148 | 0.052 | 0.389 | 0.536 | 0.010 | 1.329 | 0.256 | 0.032 |
|  | After | 0.05±0.02 | 0.11±0.07 |  |  |  |  |  |  |  |  |  |
| Gait cycle variability  (%) | Before | 0.04±0.01 | 0.05±0.04 | 1.952 | 0.170 | 0.047 | 0.342 | 0.562 | 0.008 | 0.578 | 0.452 | 0.014 |
|  | After | 0.03±0.01 | 0.05±0.05 |  |  |  |  |  |  |  |  |  |
| Double support variability (%) | Before | 0.20±0.06 | 0.16±0.05 | 3.938 | 0.054 | 0.090 | 2.390 | 0.130 | 0.056 | 0.641 | 0.428 | 0.016 |
|  | After | 0.16±0.05 | 0.14±0.05 |  |  |  |  |  |  |  |  |  |
| Arm ROM maximum variability (%) | Before | 0.15±0.10 | 0.16±0.10 | 0.695 | 0.409 | 0.017 | 0.544 | 0.465 | 0.013 | 0.066 | 0.798 | 0.002 |
|  | After | 0.12±0.04 | 0.15±0.09 |  |  |  |  |  |  |  |  |  |
| Turning average duration variability (%) | Before | 0.10±0.11 | 0.07±0.12 | 0.010 | 0.923 | 0.000 | 0.324 | 0.572 | 0.008 | 0.401 | 0.530 | 0.010 |
|  | After | 0.09±0.13 | 0.12±0.16 |  |  |  |  |  |  |  |  |  |
| Turing average angular velocity variability (%) | Before | 0.10±0.11 | 0.07±0.12 | 0.010 | 0.923 | 0.000 | 0.324 | 0.572 | 0.008 | 0.401 | 0.530 | 0.010 |
|  | After | 0.09±0.13 | 0.12±0.16 |  |  |  |  |  |  |  |  |  |

*PD* Parkinson’s Disease, *taVNS* Transcutaneous auricular vagus nerve stimulation. * *p* <0.05

**Table 3. Results of two-ways ANOVA for TUG, UPDRS-Ⅲ, Tinetti balance and gait.**

| **Scale** |  | **taVNS stimulation**  **（mean±SD）** | **shame stimulation（mean±SD）** | **Group**  **(taVNS stimulation vs shame stimulation)** | | | **Condition**  **(pre-stimulus vs post-stimulus)** | | | **Interaction**  **(group × condition)** | | |
| --- | --- | --- | --- | --- | --- | --- | --- | --- | --- | --- | --- | --- |
|  |  | | | **F (1,20)** | ***p*** | **η^2^** | **F (1,20)** | ***p*** | **η^2^** | **F (1,20)** | ***p*** | **η^2^** |
| **TUG (s)** | Before | 17.03±4.86 | 14.69±3.70 | 3.756 | 0.060 | 0.086 | 0.281 | 0.599 | 0.007 | 0.055 | 0.816 | 0.001 |
|  | After | 16.21±2.92 | 14.37±2.28 |  |  |  |  |  |  |  |  |  |
| **UPDRS-Ⅲ** | Before | 18.64±10.36 | 14.64±3.17 | 0.628 | 0.433 | 0.015 | 1.907 | 0.175 | 0.046 | 1.439 | 0.237 | 0.035 |
|  | After | 13.45±6.99 | 14.27±3.35 |  |  |  |  |  |  |  |  |  |
| **Tinetti balance** | Before | 15.55±0.69 | 15.36±1.21 | 0.592 | 0.446 | 0.015 | 0.024 | 0.878 | 0.001 | 0.024 | 0.878 | 0.001 |
|  | After | 15.64±0.67 | 15.36±1.21 |  |  |  |  |  |  |  |  |  |
| **Tinetti gait** | Before | 10.55±0.93 | 10.82±1.08 | 0.031 | 0.860 | 0.001 | 0.283 | 0.598 | 0.007 | 1.541 | 0.222 | 0.037 |
|  | After | 11.00±0.45 | 10.64±0.81 |  |  |  |  |  |  |  |  |  |

*ANOVA* Analysis of Variance, *TUG* Time Up and Go, *UPDRS* Unified Parkinson’s Disease Rating Scale, *taVNS* transcutaneous auricular Vagus Nerve Stimulation, *PD* Parkinson’s Disease.
